# Validation of a Survey Questionnaire on Organ Donation: An Arabic World Scenario

**DOI:** 10.1155/2018/9309486

**Published:** 2018-02-08

**Authors:** Rajvir Singh, Tulika Mehta Agarwal, Hassan Al-Thani, Yousuf Al Maslamani, Ayman El-Menyar

**Affiliations:** ^1^Cardiology Research Center, Heart Hospital, Hamad Medical Corporation (HMC), P.O. Box 3050, Doha, Qatar; ^2^Trauma & Vascular Surgery, Hamad Medical Corporation (HMC), P.O. Box 3050, Doha, Qatar; ^3^Hamad General Hospital (HGH), P.O. Box 3050, Doha, Qatar

## Abstract

**Objective:**

To validate a questionnaire for measuring factors influencing organ donation and transplant.

**Methods:**

The constructed questionnaire was based on the theory of planned behavior by Ajzen Icek and had 45 questions including general inquiry and demographic information. Four experts on the topic, Arabic culture, and the Arabic and English languages established content validity through review. It was quantified by content validity index (CVI). Construct validity was established by principal component analysis (PCA), whereas internal consistency was checked by Cronbach's Alpha and intraclass correlation coefficient (ICC). Statistical analysis was performed by SPSS 22.0 statistical package.

**Results:**

Content validity in the form of S-CVI/Average and S-CVI/UA was 0.95 and 0.82, respectively, suggesting adequate relevance content of the questionnaire. Factor analysis indicated that the construct validity for each domain (knowledge, attitudes, beliefs, and intention) was 65%, 71%, 77%, and 70%, respectively. Cronbach's Alpha and ICC coefficients were 0.90, 0.67, 0.75, and 0.74 and 0.82, 0.58, 0.61, and 0.74, respectively, for the domains.

**Conclusion:**

The questionnaire consists of 39 items on knowledge, attitudes, beliefs, and intention domains which is valid and reliable tool to use for organ donation and transplant survey.

## 1. Introduction

Shortage of organs for transplantation is essentially a universal and global problem [1]. Lack of knowledge, attitudes, education, gender, occupation, bodily concerns, sociodemographic concerns, community and family beliefs and values, uncertainties regarding religious permissibility, conflict between one's own desire and family values, and desire for reciprocal benefits are some of the reasons that impact an individual's organ donation decision and act as barriers to the process which have been researched by America, Europe, and other countries in the world [2, 3].

Qatar is a small country in the Arab world which has a population of 2.9 million (2016) consisting of less than 40% Qatari and Middle-Eastern Arabs. Organ donation is still in early stages of development in Qatar where challenges for organ donation and transplant are unique due to diverse socioeconomic and multiethnic population. Previous survey about organ donation limited the population to health care centers, health system, and health education, and lacks of a suitable study since the launch of the Organ Transplant and Organ Donation center in 2011 and 2012 are also found to be the main limitations of the previous survey about factors affecting organ donation in Qatar [4].

Different factors impact organ donation behavior of individuals. Such factors have not been studied within the country. Therefore, a study to explore factors that impact organ donation behavior with regard to live as well as deceased donors in Qatar through well-designed questionnaire which suites the culture and language of the residents of the country is needed.

## 2. Methodology

**Figure 1:**
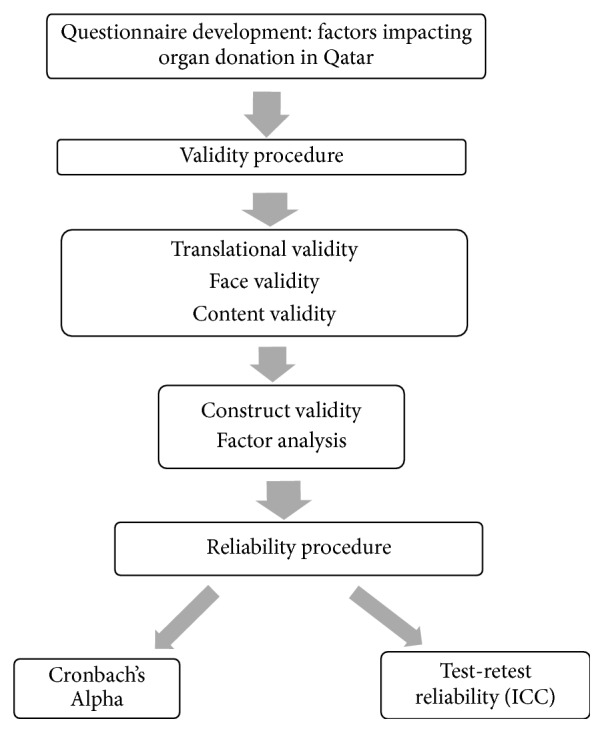
A flowchart depicting the process used for validating the questionnaire for understanding factors impacting organ donation in Qatar.

Ten steps in three phases described by Alenka Slavec and Mateja Drnovesek [5] were used for questionnaire development and validation. In Phase 1, theoretical importance and existence of the questionnaire construct were performed by three steps: content domain specification, item pool generation, and content validity evaluation. In Phase 2, representativeness and appropriateness of data collection were assessed by questionnaire development and evaluation, translation and back translation, pilot study, and data collection steps, whereas in phase 3, statistical analysis and statistical evidence of the construct were assessed by construct validity and reliability assessment of questionnaire items (Figure 1).

A pool of potential items was selected following a multistep process including an extensive review of relevant research papers [6–10] and measures both within the Arab world as well as in other parts of the world interviews with experts working in the field of organ donation or organ transplant and understanding of the theory of planned behavior [11]. In the initial phase of designing the questionnaire, items were reduced from 64 to 45 following discussions with the experts in the field of organ donation (Supplementary Materials 1 and 2). This was done by removing the items that were duplicated or lacked relevance to the country's law or cultural sensitivity. Some items were clubbed together in single question. Empirically, the “best” (i.e., most representatives, nonredundant) items were identified to assess the common underlying themes (based on theory of planned behavior). This was done for ensuring comprehensiveness and accuracy of the questionnaire.

Most of the questions in the questionnaire are closed ended and had fixed responses. However, some questions had the option of giving answers other than those that are available in the questionnaire enlisted under the title “Others (specify).” This questionnaire included sections to assess the individuals' knowledge, attitude, belief (behavioral beliefs, normative beliefs, and control beliefs), and intentions based on theory of planned behavior (Supplementary Materials 1 and 2). The initial version of the questionnaire was peer-reviewed and refined for validation.

Flesch Reading Ease score and Kincaid Grade Level were calculated to ascertain the understandability of the questionnaire using Microsoft Word 10 proofing tool for spelling and grammar.

### 2.1. Coding of Items in the Questionnaire

The questionnaire was divided into 6 domains about organ donation named general inquiry; knowledge; attitudes; beliefs; intention; and demographic information. Each domain had multiple items and subitems (Supplementary Materials 1 and 2). Each item was coded “1” and “0” for yes and no or correct and wrong response to the dichotomous questions. Items having categories such as yes, no/do not know, and maybe in the questionnaire were coded as 2, 0, and 1. Five-level Likert scale, “strongly disagree,” “disagree,” “neither agree nor disagree,” “agree,” and “strongly agree,” was coded as “−2,” “−1,” “0,” “+1,” and “+2,” respectively [12]. Negatively keyed items were given reverse code. Domain score was calculated summing up all feasible items in the domain named index.

### 2.2. Translational Validity

The resident population of Qatar constitutes most Arabic speaking individuals. The survey questionnaire was translated into Arabic by using professional translation service, so as to ensure that the translation not only communicated meaning but was also tied to local literary forms and was culturally suitable. It was also translated by one of the investigators of the study who was well versed in English and Arabic languages. The prepared versions were compared by the project manager and discussed with the translators to come up with a proofed version. Back translation of the proofed version of the questionnaire was done for ensuring the accuracy. A new harmonized translated version was prepared for face and content validity.

### 2.3. Face Validity

Four bilingual experts who had good experience in the topic were identified to ascertain whether the content of the questionnaires (English and Arabic) was relevant to the study purpose and appropriate for the prevailing culture in the Arab world. The experts were able to evaluate whether the questions successfully capture the intended topic of the survey. They also assessed question construction and common errors such as leading, confusing, or double-barreled questions. The questionnaire was assessed for clarity and accuracy as a questionnaire to assess factors influencing organ donation and organ transplant, accuracy of the presentation of the subsections within the questionnaire, suitability within cultural and legal context and clarity of language used, appropriateness for a face to face household survey, comprehensiveness for a readability and feasibility of the survey questionnaire, communication of right message, and consistency of style and formatting.

### 2.4. Content Validity

Content validity for questionnaire was assessed by computing content validity indices based on experts' rating of item relevance ((1) not relevant, (2) somewhat relevant, (3) quite relevant, and (4) highly relevant). I-CVI (individual level content validity), S-CVI/Average (scale level content validity index with average method), and S-CVI/UA (scale level content validity with universal average method) were calculated as they were advantageous with regard to each of computation, understandability, and focus on agreement of relevance [13]. Changes were made in the questionnaire based on the suggestions of the experts to reach a final version, which could be taken up for the study.

### 2.5. Construct Validity

A factor loading scale runs between −1.0 and 1.0. Principal Component Analysis (PCA) for assessing factor loading in the four domains (knowledge, attitudes, beliefs, and intention) was used to cluster items into common factors which interpret each factor according to the items loading on it and describe the items into a small number of factors (latent variables) [14]. Initial total eigenvalue reflects the number of extracted factors whose sum should be equal to number of items, which are subject to factor analysis. The higher the eigenvalue of loading, the more the factor contribution. Percentage of variance is explained by each factor and cumulative percentage is cumulative variance of the factor when added to the previous factor. Varimax rotation is used to maximize the sum of the variance. Total rotation sum of squared (squared correlation between variables and factors) loading describes variance after rotation attributable to each factor. Scree plot graph is another useful tool to determine how many factors to retain and where the curve starts to flatten. Scree plot shows the eigenvalues on the *y*-axis and the number of factors on the *x*-axis. Kaiser-Meyer-Olkin (KMO) values were considered for measuring sampling adequacy for each factor analysis.

### 2.6. Reliability

Test-retest reliability was estimated by administering the same questionnaire to the same subjects twice in the gap of 15 to 18 days [15] as a test-retest measure under an assumption that there will be no substantial change between the two scores at the two points of time [16]. Data was collected by trained research assistants hired for the research. Participants were well versed in both Arabic and English languages.

### 2.7. Sample Size

A sample of 5–50 subjects from the same sample frame was found sufficient to test the questionnaire [17]. A sample of 50 respondents using convenience sampling method has been taken for the validation of the questionnaire from the same population as that of the main study.

### 2.8. Exclusion Criteria

Domestic helpers and drivers working for and living within the household were excluded from the survey since they were not considered members of the household. As per the definition of ministry of development planning and statistics, Qatar household is defined as “one or more individuals, living together in one house, sharing food and beverage and other living aspects, in a way to form one living unit (household) which spends on its needs (goods and services) from its accumulated cash revenue, the source being one or more individuals of the household” [18]. Vulnerable population was also excluded from the study. Vulnerable population in the present study is based on guidelines offered by the Supreme Council of Health; Qatar constitutes vulnerable category of subjects in research, such as children, prisoners, pregnant women, or mentally disabled persons. These are regularly reviewed by the Institutional Review Board (IRB) [19].

### 2.9. Consent

Written consent was obtained from all the participants after sharing with them the relevant information with regard to the study, confidentiality of their identity, and how the data would be used. A standardized information sheet that included all these details was read out to the participants before consent.

### 2.10. Statistical Analysis

Two research team members who worked together while entering coded data into the spreadsheets and checking the accuracy of the data entered cleaned the collected data for analysis. Once prepared, the spreadsheets were checked once again for accuracy.

Felsch Reading Ease score and Felsch Kincaid Grade Level were calculated for readability and ease of understandability of the questionnaire. Demographic variables were summarized in terms of frequency and percentages. Content validity was performed to see relevance of the questionnaire. Construct validity was measured in the form of factor analysis using principal factor method with Varimax rotation method to test the hypothesized domain structure [20]. Kaiser-Meyer-Olkin (KMO) value of 0.8 and above was used to ensure appropriate sample size for factor analysis. The Kaiser criterion to select factors having eigenvalue ≥1 and scree plot to depict the descending variances for factors extraction in the form of graph have been presented. Eigenvalue 1 and above is considered to explain at least the same amount of variance as a single variable into factor analysis. Internal consistency and reproducibility were performed for reliability of the questionnaire. Cronbach's *α* coefficient was used to see homogeneity of question items in each domain index for internal consistency. Coefficient 0.7 and above is considered to be internally consistent for the questionnaire [21]. Each domain score in the form of index variable at pre- and postlevel was also calculated using intraclass correlation [22]. *P* value 0.05 (two-tailed) is considered as significant level. SPSS 22.0 statistical package is used for the analysis.

## 3. Results

Table 1 describes frequency and percentages of the demographic and general characteristics of the subjects. All the 50 subjects were more than 18 years of age and had resident permit of Qatar or were Qatari citizen. Male to female ratio was 22 : 28. 86% participants of the survey were non-Qatari. Most of them, 37 (74%), had educational level of higher secondary and graduation. 49 out of 50 (98%) had heard the term organ donation. Word of mouth (76%), newspaper (58%), and television (52%) were found to be prominent facilities for hearing about organ donation. Only 10% of the participants had attended organ donation promotion campaigns in Qatar before the interview. 42 (84%) subjects said that meaning of organ/tissue/blood was either transfer of tissues or organ from dead body or transfer of tissues/blood/organs from a living donor to a patient in need. 47 (94%) subjects agreed that kidney can be donated, whereas number and percentages of participants who agreed that blood, heart, liver, cornea of eyes, and bone marrow could be donated were 43 (86%), 31 (62%), 34 (68%), and 36 (72%), respectively. Coding and description items of the questionnaire are given in Table 2.

Readability statistics were calculated for the questionnaire to ascertain the understandability of the questionnaire for people of different educational levels. The Felsch Reading Ease score of the questionnaire was 69.9 which indicated that the language used in the questionnaire was plain English which could be easily understood by 13- to 15-year-old students. Felsch Kincaid Grade Level for the questionnaire was 5.6, which also indicated that the questionnaire would be understandable for anyone with a grade five education and above.

Content validity was measured for comprehensiveness and representativeness of the content of a scale. A 4-point content validity index (CVI) on behalf of judgement of 4 experts was evaluated in the form S-CVI/Average and S-CVI/UA indices. Our study showed value 0.95 for S-CVI/Average and value 0.82 for S-CVI/UA, whereas total agreement was 32 for 39 items suggesting adequate questionnaire relevance.

**Figure 2:**
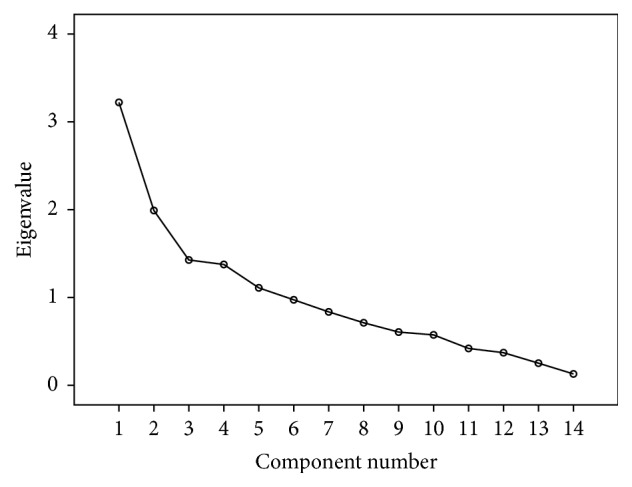
Scree plot for knowledge domain: construct validity.

**Figure 3:**
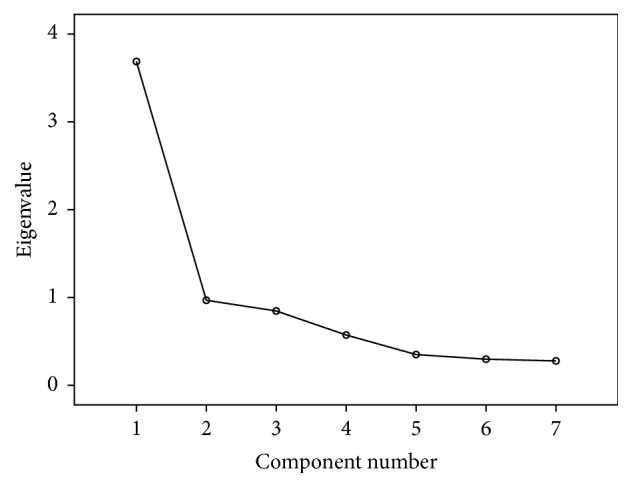
Scree plot for attitude domain: construct validity.

**Figure 4:**
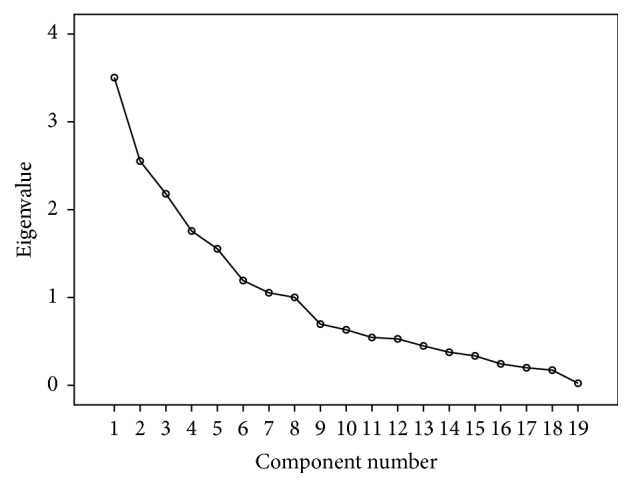
Scree plot for beliefs domain: construct validity.

**Figure 5:**
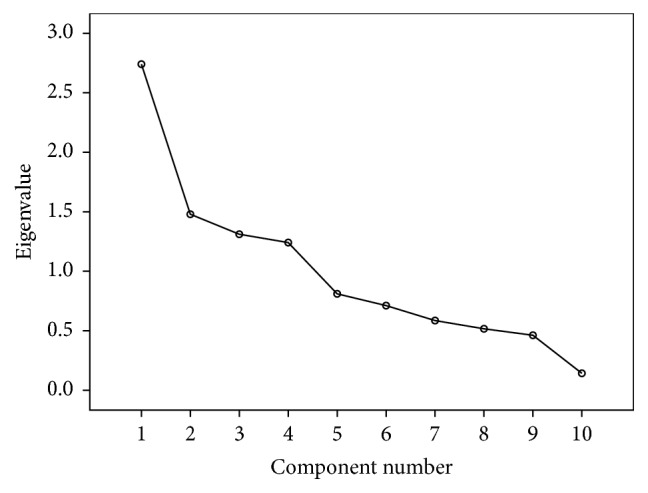
Scree plot for intention domain: construct validity.

Factor analysis was used for determining the construct validity for each domain (knowledge, attitudes, beliefs, and intention) separately. Kaiser-Meyer-Olkin (KMO) values were ≥0.80 for all the four domains. Total variance explained for knowledge, attitude, beliefs, and intension domains was 65%, 71%, 77%, and 70%, respectively. It was demonstrated by scree plots also. Results suggested good construct accuracy of the questionnaire (Tables 3, 4, 5, and 6 and Figures 2, 3, 4, and 5).

Cronbach's Alpha was used to assess factor loading and check whether questions within a given section point back to the same elements and load into the same factors. The scores obtained for all the items within the four sections of knowledge, attitude, belief, and intention on Cronbach's Alpha were 0.90, 0.67, 0.75, and 0.74, respectively, and fell under acceptable limit of 0.60–1.00 in turn indicating a good level of internal consistency within the different domains (Table 7).

Intraclass correlation coefficient (ICC) was calculated for knowledge, attitude, belief, and intention sections using the scores obtained in the pre- and postmeasures. The correlation coefficient scores being 0.82, 0.58, 0.61, and 0.74 for the four scales were all found to be highly significant at 0.001 levels suggesting adequate test-retest validity of the questionnaire (Table 7).

## 4. Revising the Survey

The survey was slightly revised based on the information gathered from the experts for grammar and spellings of the questionnaire before collecting data.

## 5. Discussion

The questionnaire was based on theory of panned behavior by Ajzen Icek which was developed and validated as a new tool for measuring factors influencing organ donation and transplantation in Arabic world scenario for the first time. Overall results demonstrated that questionnaire is an accurate measure of organ donation and transplant survey. The process of questionnaire validation is rigorous where face and content validity helped to assess whether content of questionnaire was relevant to the survey. Factor analysis also justified for theoretical construct of the questionnaire. Internal consistency was on recommended level, whereas test-retest indicated stability of the survey use over time. Therefore, organ donation and transplant questionnaire are useful for the survey.

## 6. Conclusion

Organ donation and transplant questionnaire is valid and reliable tool to use in Arabic and other wider range of population; however, it is recommended that confirmatory factor analysis on larger sample may be useful for the generalizability.

## 7. Limitations

Only four experts had been recruited in the present study for content validation. Content validity is usually done by 7 or more experts as suggested by some studies [13, 14]. However, in one of the published studies, three experts also had been recruited for evaluating content validity [23]. Convenience sampling method has been used for data collection, where nationality was controlled according to distribution of the population. Calculated indices in the study could not be compared as no related review of literature was available on validation of the organ donation questionnaire. Total variance explained is the most important in development of a questionnaire; hence, items loadings for each component for each factor analysis have not been described in tables.

## Figures and Tables

**Table 1:** Demographic and general characteristics.

Variables	Category	Number (%)
Age in years	≥18	50 (100)
Gender	Male/female	22 (44)/28 (56)
Nationality	Qatari citizen/non-Qatari resident	7 (14)/43 (86)
Income/month	QR < 10,000	10 (20)
	QR 10,000 to 20,000	25 (50)
	QR 20100 to 30000	6 (12)
	QR > 30000	1 (2)
	Refused	8 (16)
Education	Primary	3 (6)
	Secondary	5 (10)
	Higher secondary	11 (22)
	Graduation	26 (52)
	Postgraduation and above	3 (6)
	Others	1 (2)
Heard term organ donation	Yes	49 (98)
Heard about organ donation by word of mouth	Yes	38 (76)
Heard about organ donation through newspaper	Yes	27 (58)
Heard about organ donation through television	Yes	26 (52)
Attended organ donation campaign	Yes	5 (10)
Know about donation of kidney	Yes	47 (94)
Know about donation of blood	Yes	43 (86)
Know about donation of heart	Yes	31 (62)
Know about donation of liver	Yes	34 (68)
Know about donation of cornea	Yes	36 (72)

**Table 2:** Description of questionnaire items/constructs.

Knowledge about organ donation	Number of items	Descriptions

What does organ/tissue/blood donation mean to you?	1	Transfer of tissues or organ from a dead body to a patient; transfer of tissues or organ from a dead body to a patient in need; transfer of tissues/blood/organs from a living to a patient in need; all the above (coded: 1, 2, 3, 4).

What organs/tissues can be donated?	12	Kidney; heart; liver; lungs; pancreas; intestine; blood; cornea of the eyes; skin; bone marrow; bone; all the above (coded: yes = 1/no = 0).

There is a donor registry in Qatar where people register during their life to donate organs after death	1	Have you heard about it (coded: yes = 1/no = 0).

At what age can an individual register for organ donation?	3	At any age; 18 years and above; do not know (coded: correct = 1/incorrect = 0).

Death could mean	1	The heart is not beating and there is no breathing; brain death in which the heart is beating with the help of ventilator to keep breathing; I do not know; other (coded: correct = 1/incorrect = 0).

Does your religion allow organ donation?	1	Religion allow: (coded: yes = 1/no = 0/do not know = 0).

Do you know anyone who has donated an organ?	1	Family member; friend; colleague; no one; other (coded: yes = 1/no = 0).

Do you know that during life an individual can donate a part of his liver to his relative?	1	Can donate part of liver (coded: yes = 1/no = 0).

Do you know that donating a part of your liver is a risk to your health?	1	Donating part of liver is a risk (coded: yes = 2/no = 0/may be = 1/do not know = 0).

Do you know that you can donate one of your two kidneys during your life, to another person?	1	Can donate one kidney during your life (coded: yes = 1/no = 0).

Do you know that donating a kidney is safe?	1	Donating kidney is safe (coded: yes = 2/no = 0/may be = 1/do not know = 0).

Do you know the Qatar organ donation law and policy	6	Prohibits any buying or selling of organs; provides access to transplant facility for all nationalities equally; gives donated organs from deceased donors to the first person on the waiting list regardless of nationality; puts no pressure on the deceased donor family or living donor to donate; all live donors in Qatar are provided with health insurance for life; all families of the deceased in Qatar will receive social support if they need it (coded: yes = 1/no = 0).

Attitudes	Number of items	Descriptions

Organ donation is a good thing and should be promoted	1	Agreement with (coded: strongly agree = +2 to strongly disagree = −2).

Registering as organ donor could save somebody's life	1	Agreement with (coded: strongly agree = +2 to strongly disagree = −2).

Qatari as well as non-Qatari residents should be automatically included on the organ donor register of Qatar, with the ability to refuse if they wish	1	Agreement with (coded: strongly agree = +2 to strongly disagree = −2).

I am willing to register as an organ donor, if my family would have no objection to allowing donation of my organs at the time of my death	1	Agreement with (coded: strongly agree = +2 to strongly disagree = −2).

If I knew more about what is organ transplant and how it is done	1	Agreement with (coded: strongly agree = +2 to strongly disagree = −2).

If more information was available about the viewpoint of my religion with regard to organ donation	1	Agreement with (coded: strongly agree = +2 to strongly disagree = −2).

If I knew where I could register	1	Agreement with (coded: strongly agree = +2 to strongly disagree = −2).

Behavioral beliefs	Number of items	Descriptions

I think my donation whether living or after death is going to impact my life after death in a good way	1	Agreement with (coded: strongly agree = +2 to strongly disagree = −2).

Organ donation is an act which will be rewarded by God	1	Agreement with (coded: strongly agree = +2 to strongly disagree = −2).

In case of an emergency, doctors will not provide enough care if the patient is registered organ donor	1	Agreement with (coded: strongly agree = +2 to strongly disagree = −2).

Organ retrieval process after death may cause body disfigurement	1	Agreement with (coded: strongly agree = +2 to strongly disagree = −2).

Organ donation will increase if social support is provided to family of the deceased regardless of whether they donate or not	1	Agreement with (coded: strongly agree = +2 to strongly disagree = −2).

Normative beliefs/subjective norms	Number of items	Descriptions

To register as an organ donation, you will take the opinion	6	Family member; my community; religious leader; friend; other (yes/no).

Control beliefs/perceived behavioral control	Number of items	Descriptions

You do not find many opportunities to register as organ donor in Qatar	1	Agreement with (coded: strongly agree = +2 to strongly disagree = −2).

Organ donor registration is time consuming process (asked only if registered in Qatar)	1	Agreement with (coded: strongly agree = +2 to strongly disagree = −2).

While registering for organ donation, you may not get answer for all your questions	1	Agreement with (coded: strongly agree = +2 to strongly disagree = −2).

You are not healthy to donate	1	Agreement with (coded: strongly agree = +2 to strongly disagree = −2).

Your age is not fit for donating your organ donation	1	Agreement with (coded: strongly agree = +2 to strongly disagree = −2).

Operation procedure for procuring organs is discouraging	1	Agreement with (coded: strongly agree = +2 to strongly disagree = −2).

Live donation	Number of items	Descriptions

You are worried that organ donation might leave you weak and disabled	1	Agreement with (coded: strongly agree = +2 to strongly disagree = −2).

I do not trust the health care system in Qatar and it is better to go abroad for organ donation and organ transplantation	1	Agreement with (coded: strongly agree = +2 to strongly disagree = −2).

Donation after death	Number of items	Descriptions

Emotions of your family members while organs are being taken make you feel concerned	1	Agreement with (coded: strongly agree = +2 to strongly disagree = −2).

Intentions to Organ donation	Number of items	Descriptions

Are you willing to register as an organ/tissue donor	1	Willingness (yes/no).

Which organ/tissue will you prefer to donate?	1	Kidney; blood; heart; eyes; liver; skin; lungs; bone marrow; all the above (yes/no).

Do you have religious leader who you trust?	1	Religious leader (yes/no).

Would you consider organ donation after discussion with a religious leader?	1	Discussion with religious leader (yes/no).

Would you consider donating organ more seriously if you are approached by an organization you could trust	1	Agreement with (coded: strongly agree = +2 to strongly disagree = −2).

*Note*. (1) Questionnaire also includes questions on demographic variables, age, known language, gender, resident permit, nationality, occupation income, religion, total number of dependent family members, level of education, and duration of living in Qatar; (2) some items codes were reverted for factor analysis (PCA).

**Table 3:** Factor analysis knowledge domain: construct validity.

Component	Initial eigenvalues			Rotation sums of squared loadings
	Total	% of variance	Cumulative%	Total
1	3.221	23.006	23.006	2.594
2	1.990	14.216	37.222	1.960
3	1.427	10.191	47.414	1.936
4	1.376	9.827	57.240	1.978
5	1.110	7.928	65.168	1.550

**Table 4:** Factor analysis attitude domain: construct validity.

Component	Initial eigenvalues			Rotation sums of squared loadings
	Total	% of variance	Cumulative%	Total
1	2.314	33.060	33.060	2.105
2	1.490	21.292	54.35	1.550
3	1.183	16.90	71.255	1.672

**Table 5:** Factor analysis beliefs domain: construct validity.

Component	Initial eigenvalues			Rotation sums of squared loadings
	Total	% of variance	Cumulative%	Total
1	3.503	18.438	18.438	3.106
2	2.553	13.463	31.874	2.157
3	2.180	11.475	43.349	2.343
4	1.757	9.249	52.597	1.904
5	1.554	8.179	60.777	1.807
6	1.193	6.280	67.056	1.748
7	1.054	5.545	72.601	1.697
8	1.001	5.268	77.869	1.496

**Table 6:** Factor analysis intention domain: construct validity.

Component	Initial eigenvalues			Rotation sums of squared loadings
	Total	% of variance	Cumulative%	Total
1	2.804	28.042	28.042	2.078
2	1.727	17.274	45.316	2.008
3	1.383	13.829	59.145	1.756
4	1.130	11.301	70.445	1.203

**Table 7:** Internal consistency and test-retest validity of questionnaire domains indices.

Variable	Cronbach's Alpha	Intraclass correlation
Knowledge towards organ donation index	0.90	0.82
Attitudes towards organ donation index	0.67	0.58
Belief towards organ donation index	0.75	0.61
Intention towards organ donation index	0.74	0.74

*Note*. Cronbach's Alpha acceptable limit: 0.60 to 0.70. Above is most favorable.

## Supplementary Materials

Supplementary 1 Questionnaire in English.

Supplementary 2 Questionnaire in Arabic.
