# Supplementary material for: Validation of a Survey Questionnaire on Organ Donation: An Arabic World Scenario
Source: J Transplant. 2018 Feb 8;2018:9309486. doi: 10.1155/2018/9309486 (PMC5822804; doi:10.1155/2018/9309486)
Supplement: Supplementary 1 — Questionnaire in English. [file 9309486.f1.pdf]

Factors Influencing Organ Donation in Qatar

SETTING THE CRITERIA

1. May I know the language of your choice for the interview?

   Language _________

2. Age: Are you 18 or over 18
   a. Yes
   b. No (Terminate)
   c. Don’t Know (Terminate)
   d. Refused (Terminate)

3. Do you have a Qatari resident permit/ Qatari Citizenship

a. Yes [Continue]
b. No [Terminate]
c. Under process [Continue]

Survey Questionnaire

Participant Serial #:

Section 1: General Inquiry

1. Have you ever heard of the term "Organ Donation"?

a. Yes b. No

(If 'No', Skip Q2, & Q3)

2. How did you hear about organ donation

a. Word of mouth
b. Newspaper
c. Television
d. Radio
e. Internet
f. Social event
g. Social get together
h. Don't know
i. Refuse
j. Other (Specify)

3. Have you ever attended organ donation promotion campaigns in Qatar

a. Yes b. No

Please Specify if 'Yes':

4. Are you registered as an organ donor?

a. Qatar b. Outside Qatar c. Not registered.

5. Have you ever donated any organ/ blood / tissues?

(If answer to question No 1 is 'No' ask only about blood or tissue donation)

a. Yes b. No

Please Specify:________________

Section 2: Knowledge about organ donation

6. What does organ/tissue/blood donation mean to you?

a. Transfer of tissues or organ from a dead body to a patient in need

b. Transfer of tissues /blood/organs from a living donor to a patient in need

c. All of the above

d. Others (specify) ____

7. What organs/tissues can be donated? (Should not be read to respondents)

a. Kidney	g Blood
b. Heart	h. Cornea of the Eyes
c. Liver	i Skin
d. Lungs	j Bone marrow .
e. Pancreas	k. Bone
f. Intestine	l All of above
m None of the above	n. Others (Please specify)_______________

8. There is a donor registry in Qatar where people register during their life to donate organs after death. Have you heard about it?

a. Yes b. No c. Partially

9. At what age can an individual register for organ donation?

a) At any age
b) 18 years and above
c) Don't know

10. Death could mean:

a. The heart is not beating and there is no breathing

b. Brain death in which the hearth is beating with the help of ventilator, to keep breathing

c. I don't know

d. Other (Please Specify)_________________

11. Does your religion allow organ donation?

a. Yes b. No c. don't know

12. Do you know anyone who has donated an organ?

a. Family member b. Friend c. Colleague d. No one
e. Others (please specify) _________________

13. Do you know that during life an individual can donate a part of his liver to his relative?

a. Yes b. No

14. Do you know that donating a part of your liver is a risk to your health?

a. Yes b. No c. Maybe d. don't know

15. Do you know that you can donate one of your two kidneys during your life, to another person?

a. Yes b. No

16. Do you know that donating a kidney is safe?

a. Yes b. No c. Maybe d. don't know

17. Do you know that the Qatar Organ Donation law and policy:

a) Prohibits any buying or selling of organs: Yes/No

b) Provides access to transplant facility for all nationalities equally: Yes/No

c) Gives donated organs from deceased donors to the first person on the waiting list regardless of nationality: Yes/No

d) Puts no pressure on the deceases donor family or living donor to donate: Yes/No

e) All Live donors in Qatar are provided with health insurance for life?

f) All families of the deceased in Qatar will receive social support if they need it: Yes/No

Section 3

Section 3.1: Attitudes

I will now be asking you some questions which will tell me about your attitude towards organ donation.

(The LIKERT SCALE: Do not read the neither agree nor disagree as response option)

18. Organ donation is a good thing and should be promoted

Strongly agree O Agree O Neither agree nor disagree O Disagree O Strongly disagree O

19. Registering as organ donor could save somebody's life

Strongly agree O Agree O Neither agree nor disagree O Disagree O Strongly disagree O

20. Qatari as well as Non Qatari residents should be automatically included on the Organ Donor register of Qatar, with the ability to refuse if they wish

Strongly Agree O Agree O Neither agree nor disagree O Disagree O Strongly disagree O

I would be more willing to register as an organ donor:

21. If I knew that my family would have no objection to allowing donation of my organs at the time of my death

Strongly Agree O Agree O Neither agree nor disagree O Disagree O Strongly disagree O

22. If I knew more about what is organ transplant and how it is done

Strongly Agree O Agree O Neither agree nor disagree O Disagree O Strongly disagree O

23. If more information was available about the viewpoint of my religion with regard to organ donation

Strongly Agree O Agree O Neither agree nor disagree O Disagree O Strongly disagree O

24. If I knew where I could register

Strongly Agree O Agree O Neither agree nor disagree O Disagree O Strongly disagree O

<u>Section 3.2: Beliefs</u>

Now I will be asking some questions to know more about your belief in relation to organ donation. These would include beliefs that decide your behavior, your belief about whose opinion influences your decisions, and what you believe can stop you from registering for donation

3.2a Behavioral Beliefs

(Beliefs that decide your behavior)

25. I think my donation weather living or after death is going to impact my life after death in a good way

Strongly Agree O Agree O Neither agree nor disagree O Disagree O Strongly disagree O

26. Organ donation is an act which will be rewarded by God

Strongly Agree O Agree O Neither agree nor disagree O Disagree O Strongly disagree O

27. In case of an emergency, doctors will not provide enough care if the patient is a registered organ donor

Strongly Agree O Agree O Neither agree nor disagree O Disagree O Strongly disagree O

28. Organ retrieval process after death may cause body disfigurement

Strongly Agree O Agree O Neither agree nor disagree O Disagree O Strongly disagree O

29. Organ donation will increase if social support is provided to family (of the deceased), regardless of whether they donate or not

Strongly Agree O Agree O Neither agree nor disagree O Disagree O Strongly disagree O

3.2b Normative Beliefs/ Subjective Norms

(Whose opinion has a strong influence on your decisions?)

30. To register as an organ donor in Qatar: you will take the opinion of

(Researcher can choose more than one option; however, options should not be read to respondents)

a. Family member (please specify)……………………..
b. My Community
c. Religious leader
d. Friend
e. No one
f. Others (please specify)______________________________

3.2c Control Beliefs/ Perceived Behavioral Control

(What you believe can stop you from registering for donation)

31. You don't find many opportunities to register as organ donor in Qatar

Strongly Agree O Agree O Neither agree nor disagree O Disagree O Strongly disagree O

32. Organ donor registration is time consuming process
(Ask only if registered in Qatar)

Strongly Agree O Agree O Neither agree nor disagree O Disagree O Strongly disagree O

33. While registering for organ donation, you may not get answer for all your questions

Strongly Agree O Agree O Neither agree nor disagree O Disagree O Strongly disagree O

34. You are not healthy to donate

Strongly Agree O Agree O Neither agree nor disagree O Disagree O Strongly disagree O

35. Your age is not fit for donating your organ.

Strongly Agree O Agree O Neither agree nor disagree O Disagree O Strongly disagree O

36. Operation procedure for procuring organs is discouraging
Strongly Agree O Agree O Neither agree nor disagree O Disagree O Strongly disagree O

Live Donation

37. You are worried that organ donation might leave you weak and disabled

Strongly Agree O Agree O Neither agree nor disagree O Disagree O Strongly disagree O

38. I don't trust the health care system in Qatar and it is better to go abroad for organ donation and organ transplantation

Strongly Agree O Agree O Neither agree nor disagree O Disagree O Strongly disagree O

Donation after Death

39. Emotions of your family members while organ are being taken make you feel concerned

Strongly Agree O Agree O Neither agree nor disagree O Disagree O Strongly disagree O

Section 4: Intentions

The next set of questions will let us understand your intentions with regard to making donation in future. (If Answer to Q4 is Qatar skip this section)

40. Are you willing to register as an Organ/Tissue donor in Qatar?

(Ask if not already registered in Qatar)

Yes No Not Decided

(If 'No' Skip 41)

41. If you are willing to donate which organs or tissue will you prefer to donate?

(Read the options to the participant. The researcher can choose more than one option. Ask only if willing)

a. Kidney b. Blood
c. Heart d. Eyes
e. Liver f. Skin
g. Lungs h. Bone marrow
j. All of above k. None of the above
l. Others (Please specify) ________________________

42. Do you have a religious leader who you trust?

a. Yes b. No

43. Would you consider organ donation after discussion with a religious leader?

a. Yes b. No c. Maybe d. Don't Know

44. Would you consider donating organ more seriously if you are approached by an organization you could trust for e.g. HMC, PHCC?

Strongly Agree O Agree O Neither agree nor disagree O Disagree O Strongly disagree O

1 Section 5: Demographic information

45. May I ask you some general questions about yourself?

i. Gender (Select yourself, don't ask):

Male/Female

ii. Age:

iii. What is your nationality?

iv. What is your occupation: (Choose the appropriate from below)

Student/ Home maker/ Government employee/ Non-government employee/ Self-

employed/Retired/ Unemployed

(Skip next if self-employed, retired or unemployed)

v. Job Sector: (Choose the appropriate from below)

Agriculture/ Petrochemical/ Manufacturing/Construction/Services/Healthcare/Transport/ Others (Please specify)……………………….

vi. Approximately what is your Household Monthly Income level in Qatari Riyal? Please consider salary and any other income e.g. rent, social welfare, etc:

(Choose the appropriate from below)

a. Less than QR10,000/month
b. QR10,000 –20,000/month
c. QR 20,100 – QR 30,000/month
d. QR 30,000 and above
e. Refused

vii. Is there more than one earning member in your household?

Yes ☐ No ☐

viii. What is your Religion: Islam /Christianity/Others (Please Specify?)

ix. Marital status: (Choose the appropriate from below):

Single /Married/ Divorced/ Widowed

x. Total number of dependent family members inside as well as outside Qatar:

xi. Level of education: (Choose the appropriate from below)

Primary (till class 5)/ Secondary (till class 10)/ higher secondary (till class 12 or equivalent) / Graduation/ Post graduation studies/ Doctoral studies/Informal education/ Diploma/ Can read and write name only/ cannot read and write

xii. How long have you been living in Qatar?

a. _______year(s) _________month(s)

b. All my life

Other Details:

a. Date of Interview

b. Interviewer Name:

c. Supervisor Name:

d. Name of Municipality:

e. Cluster No:

f. Interview Start Time:

ii. Interview End Time:

**** Thank you for your valuable time and effort. Any suggestions/opinions regarding the questionnaire and its improvement are most welcome.***

Additional Information:

Would you be willing to give consent for organ donation of your family member if approached?
Yes/ No/ Not decided

Participant Serial #:

Additional Information for Quality Check

Your Complete Name:

Contact Details:

Place of Residence in Qatar:

Name of Municipality:

Cluster No:

Residential Address (Data collector will fill herself/himself)

Other Details:

Date of Interview:

Interviewer Name:

Enumerator No:

Supervisor Name:

Interview Start Time:

Interview End Time:
