# Supplementary material for: Validation of a Survey Questionnaire on Organ Donation: An Arabic World Scenario
Source: J Transplant. 2018 Feb 8;2018:9309486. doi: 10.1155/2018/9309486 (PMC5822804; doi:10.1155/2018/9309486)
Supplement: Supplementary 2 — Questionnaire in Arabic. [file 9309486.f2.pdf]

العوامل المؤثرة على التبرع بالأعضاء في دولة قطر

وضع المعايير

1. هل لي أن أعرف اللغة التي تفضل إجراء المقابلة لها؟

   اللغة ________

2. السن: هل أنت في سن الثامنة عشر أم أكبر؟

   أ) نعم
   ب) لا (إنهاء)
   ت) لا أعلم (إنهاء)
   ث) أرفض الإجابة (إنهاء)

3. هل لديك تصريح إقامة في دولة قطر / هل أنت مواطن قطري؟

   أ) نعم (استكمال)
   ب) لا (إنهاء)
   ت) قيد المعالجة (استكمال)

مسح استقصائي

الرقم التسلسلي للمشارك:

القسم 1: سؤال عام

1. هل سمعت من قبل عن مصطلح "التبرع بالأعضاء"؟

أ) نعم ب) لا

(إذا كانت الإجابة لا، تخطى السؤال الثاني والثالث)

2. كيف سمعت عن التبرع بالأعضاء؟

أ) الدردشة مع آخرين
ب) الصحف
ت) التلفاز
ث) الراديو
ج) الإنترنت
ح) حدث اجتماعي
خ) تجمع اجتماعي
د) لا أعلم
ذ) أرفض الإجابة
ر) أخرى (يرجى التحديد)

3. هل شاركت من قبل في حملات ترويجية للتبرع بالأعضاء؟

أ) نعم ب) لا

يرجى التحديد إن كانت الإجابة "نعم":

4. هل أنت مسجل كفرد متبرع بالأعضاء؟

أ) في قطر　　　ب) خارج قطر　　　ت) غير مسجل

5. هل تبرعت من قبل بأي عضو / دم / أنسجة؟

(لو كانت إجابة السؤال رقم (1) لا، يتم السؤال عن التبرع بالدم أو الأنسجة فقط.)

أ) نعم　　　ب) لا

يرجى التحديد____________

القسم 2: المعرفة عن التبرع بالأعضاء

6. ماذا يعني لك مصطلح التبرع بالأعضاء / الدم / الأنسجة؟

أ) نقل الأنسجة أو الأعضاء من جسد المتوفى لمريض في حاجة إليها

ب) نقل الأنسجة / الدم/ الأعضاء من متبرع حي لمريض في حاجة إليها

ت) كل ما سبق أعلاه

ث) أخرى (مع التحديد) ______________________________

7. ما هي الأعضاء / الأنسجة التي يمكن التبرع بها؟ (يجب عدم قراءتها على المشارك)

أ) الكلى	د) الدم
ب) القلب	ذ) قرنية العين
ت) الكبد	ر) الجلد
ث) الرئة	ز) نخاع العظم
ج) البنكرياس	س) العظم
ح) الأمعاء	ش) كل ما سبق
خ) لا شيء مما سبق	ص) أخرى (يرجى التحدي) ________

8. يوجد سجل للتبرع في دولة قطر يسجل فيه الناس خلال حياتهم للتبرع بالأعضاء بعد الموت. هل سمعت عنه؟

أ) نعم ب) لا ت) جزئيًا

9. في أي سن يمكن للفرد التسجيل للتبرع بالأعضاء؟

أ) في أي سن

ب) 18 سنة فأكثر

ت) لا أعلم

10. الموت قد يعني:

أ) توقف دقات القلب والتنفس

ب) الموت الدماغي حيث يستمر القلب في النبض بمساعدة جهاز التنفس الصناعي

ت) لا أعلم

ث) أخرى (يرجى التحدي) ______________

11. هل يبيح الدين الذي تعتنقه التبرع بالأعضاء؟

أ) نعم ب) لا ت) لا أعلم

12. هل تربط معرفة بأي شخص تبرع بأعضائه؟

أ) أحد أفراد الأسرة ب) صديق ت) زميل ث) لا

ج) أخرى (يرجى التحدي) ______________

13. هل تعلم أن باستطاعة أي شخص التبرع خلال حياته بجزء من كبده لأحد أقاربه؟

أ) نعم ب) لا

14. هل تعلم أن تبرعك بجزء من كبدك يشكل خطورة على صحتك؟

أ) نعم ب) لا ت) ربما ث) لا أعلم

15. هل تعلم أنك تستطيع التبرع بإحدى كليتيك لشخص آخر أثناء حياتك ؟

أ) نعم ب) لا

16. هل تعلم أن التبرع بالكلية آمن؟

أ) نعم ب) لا ت) ربما ث) لا أعلم

17. هل تعلم أن القانون القطري للتبرع بالأعضاء:

أ) يحظر شراء أو بيع الأعضاء: نعم / لا
ب) يوفر الوصول إلى مرفق زراعة الأعضاء لجميع الجنسيات على حد سواء: نعم / لا
ت) يعطي الأعضاء المتبرع بها من المتوفين المتبرعين بها إلى أول شخص على قائمة الانتظار بغض النظر عن جنسيته : نعم / لا
ث) لا يضع أي ضغط على عائلة المتبرع المتوفي أو المتبرع الحي للتبرع: نعم/ لا
ج) يتمتع كل المتبرعين الأحياء في دولة قطر بتأمين صحي مدى الحياة؟
ح) جميع أسر المتوفين في قطر ستتلقى الدعم الاجتماعي إذا كانوا في حاجة إليه: نعم / لا

<u>القسم 3</u>

<u>القسم 3.1: الاتجاهات</u>

<u>سوف أوجه لك الآن بعض الأسئلة التي سوف توضح لي موقفك تجاه التبرع بالأعضاء.</u> (مقياس ليكرت: لا تقرأ خيار "لا أوافق ولا أرفض" كخيار بين الإجابات)

18. التبرع بالدم جيد ويجب دعمه

أوافق بشدة O أوافق O لا أوافق ولا أرفضO أرفض O أرفض بشدة O

19. التسجيل كمتبرع بالأعضاء قد ينقذ حياة شخص ما

أوافق بشدة O أوافق O لا أوافق ولا أرفضO أرفض O أرفض بشدة O

20. ينبغي إدراج السكان القطريين وغير القطريين تلقائيًا في سجل المتبرعين بالأعضاء في دولة قطر، مع منحهم القدرة على رفض ذلك إذا رغبوا

أوافق بشدة O أوافق O لا أوافق ولا أرفضO أرفض O أرفض بشدة O

أود أن أعرب عن استعدادي التام للتسجيل كمتبرع بالأعضاء:

21. إذا علمت أن عائلتي لن يكون لها أي اعتراض على السماح بالتبرع بأعضائي بعد وفاتي

أوافق بشدة O أوافق O لا أوافق ولا أرفضO أرفض O أرفض بشدة O

22. إذا تم إفادتي بمزيد من المعلومات عن ماهية زراعة الأعضاء وكيفية إجرائها

أوافق بشدة O أوافق O لا أوافق ولا أرفضO أرفض O أرفض بشدة O

23. إذا تم إفادتي بمزيد من المعلومات عن رأي الدين الذي اعتنقه بخصوص التبرع بالدم

أوافق بشدة O أوافق O لا أوافق ولا أرفضO أرفض O أرفض بشدة O

24. إذا علمت مكان التسجيل

أوافق بشدة O أوافق O لا أوافق ولا أرفضO أرفض O أرفض بشدة O

القسم 3.2: المعتقدات

سوف أوجه لك الآن بعض الأسئلة للتعرف أكثر على معتقدك بشأن التبرع بالأعضاء بما في ذلك المعتقدات التي تقرر سلوكك ومن هم الأشخاص الذين تعتقد أن رأيهم يؤثر على قراراتك وما هي الأشياء التي تعتقد أنها قد تمنعك من التسجيل

3.2أ المعتقدات السلوكية

(المعتقدات التي تقرر سلوكك)

25. اعتقد أن تبرعي بالأعضاء سواء كنت حيًا أو ميتًا سيكون له تأثير إيجابي على حياتي بعد الموت

أوافق بشدة O   أوافق O   لا أوافق ولا أرفضO   أرفض O   أرفض بشدة O

26. التبرع بالأعضاء من الأعمال التي سيجازيني الله عليها خيرًا

أوافق بشدة O   أوافق O   لا أوافق ولا أرفضO   أرفض O   أرفض بشدة O

27. في حالات الطوارئ، لن يجد المريض العناية الكافية من الأطباء إذا كان مسجلاً كمتبرع بالأعضاء

أوافق بشدة O   أوافق O   لا أوافق ولا أرفضO   أرفض O   أرفض بشدة O

28. قد يتسبب أخذ الأعضاء بعد الموت في تشوهات جسدية

أوافق بشدة O   أوافق O   لا أوافق ولا أرفضO   أرفض O   أرفض بشدة O

29. سوف يزيد التبرع بالأعضاء في حالة توفير الدعم الاجتماعي لأسرة المتوفي، بصرف النظر عن قرارهم بالتبرع من عدمه

أوافق بشدة O   أوافق O   لا أوافق ولا أرفضO   أرفض O   أرفض بشدة O

3.2.ب المعتقدات المعيارية / المعايير الشخصية

(من الشخص الذي تعتقد أن رأيه له تأثير قوي على قراراتك؟)

30. للتسجيل كمتبرع بالأعضاء في دولة قطر: سوف تأخذ برأي:

(يمكن للباحث اختيار أكثر من اختيار إلا ان هذه الخيارات لا يجب قراءتها للمشارك في المسح)

أ) أحد أفراد الأسرة......................
ب) مجتمعي
ت) مرشد ديني
ث) صديق
ج) لا أحد
ح) آخرين (يرجى التحديد) ____________________________

3.2.ت معتقدات التحكم/ الضوابط السلوكية للتصور

(ماهي الأشياء التي تعتقد أنها قد تمنعك من التسجيل للتبرع)

31. لا تتاح أمامك فرص عديدة في دولة قطر للتسجيل كمتبرع بالأعضاء

أوافق بشدة O أوافق O لا أوافق ولا أرفضO أرفض O أرفض بشدة O

32. عملية التسجيل كمتبرع بالأعضاء تستغرق وقتًا طويلًا

(يوجه هذا السؤال للمسجلين كمتبرعين في قطر فقط)

أوافق بشدة O أوافق O لا أوافق ولا أرفضO أرفض O أرفض بشدة O

33. أثناء التسجيل للتبرع بالأعضاء، قد لا تتلقى إجابات عن كافة الأسئلة

أوافق بشدة O أوافق O لا أوافق ولا أرفضO أرفض O أرفض بشدة O

34. لا تتمتع بالصحة للتبرع

أوافق بشدة O أوافق O لا أوافق ولا أرفضO أرفض O أرفض بشدة O

35. عمرك لا يناسب التبرع بالأعضاء

أوافق بشدة O أوافق O لا أوافق ولا أرفضO أرفض O أرفض بشدة O

36. إجراءات عملية شراء الأعضاء غير مشجعة

أوافق بشدة O أوافق O لا أوافق ولا أرفضO أرفض O أرفض بشدة O

التبرع أثناء الحياه

37. تخشى من أن التبرع بالدم قد يجعلك ضعيف وعاجز

أوافق بشدة O أوافق O لا أوافق ولا أرفضO أرفض O أرفض بشدة O

38. لا أثق في نظام الرعاية الصحية بدولة قطر وأفضل السفر للخارج للتبرع بالأعضاء وزراعتها

أوافق بشدة O أوافق O لا أوافق ولا أرفضO أرفض O أرفض بشدة O

التبرع بعد الوفاه

39. مشاعر أفراد أسرتك أثناء أخذ الأعضاء قد تشعرك بالقلق من الإقدام على هذا الفعل

أوافق بشدة O　　أوافق O　　لا أوافق ولا أرفضO　　أرفض O　　أرفض بشدة O

القسم 4: النوايا

مجموعة الأسئلة التالية ستدعنا نفهم نواياك فيما يتعلق بالتبرع في المستقبل.

(لو كانت اجابة السؤال رقم ٤ (قطر) تخطي هذا القسم من الاسئلة)

40. هل لديك استعداد للتسجيل كمتبرع بالأعضاء / الأنسجة في قطر؟

((يوجه هذا السؤال لغير لمسجلين كمتبرعين في قطر)

نعم　　لا　　لم أقرر بعد

(إذا كانت الإجابة بلا فتخطى السؤال 40)

41. إذا كان لديك استعداد للتبرع فما هي الأعضاء / الأنسجة التي تفضل التبرع بها؟

(عليك قراءة الخيارات للمشارك ويمكن للباحث اختيار أكثر من خيار واحد)

أ) الكلى　　د) الدم

ب) القلب　　ذ) العين

ت) الكبد　　ر) الجلد

ث) الرئة　　ز) نخاع العظم

ج) جميع ما سبق　　س) لا شيء مما سبق

ح) أخرى (يرجى التحدي) _______

42. هل تثق في مرشد ديني معين؟

أ) نعم　　ب) لا

(إذا كانت الإجابة بـ(لا) أو (لم أقرر بعد) فتخطى السؤال 41)

43. هل ستدرس القيام بالتبرع بالأعضاء بعد مناقشة مرشدك الديني؟

أ) نعم ب) لا ت) ربما ث) لا أعلم

44. هل ستدرس التبرع بالأعضاء بشكل أكثر جدية إذا تم الاتصال بك من مؤسسة تثق بها كمؤسسة حمد الطبية أو مؤسسة الرعاية الصحية الأولية؟

أوافق بشدة O أوافق O لا أوافق ولا أرفضO أرفض O أرفض بشدة O

القسم 5: المعلومات الديموغرافية

45. هل تسمح لي بأن أوجه لك بعض الأسئلة العامة عنك؟

أ) النوع (اختاره بنفسك ولا داعي للاستفسار)

ذكر/ أنثى

ب) العمر:

ت) ما هي جنسيتك؟

ث) ما هي مهنتك: (اختر الإجابة المناسبة من الخيارات الواردة أدناه)

طالب / ربة منزل/ موظف حكومي/موظف غير حكومي / أعمل لحسابي الخاص / متقاعد / عاطل

(تخطى السؤال التالي إذا كانت الإجابة لحسابي الخاص أو متقاعد أو عاطل)

ج) قطاع العمل: (اختر الإجابة المناسبة من الخيارات الواردة أدناه)

الزراعة / البتروكيماويات / الصناعة التحويلية / البناء / الخدمات / الرعاية الصحية / النقل والمواصلات / أخرى (يرجى تحديدها) ....

ح) ما هو تقريبًا مستوى دخل الأسرة الشهري بالريال القطري؟ يرجى مراعاة الرواتب و أي دخل آخر كالإيجارات والرعاية الاجتماعية، الخ :

(اختر الإجابة المناسبة من الخيارات الواردة أدناه)

1) أقل من 10,000 ريالًا قطريًا في الشهر

2) 10,000 - 20,000 ريالًا قطريًا في الشهر

3) 20,100 - 30,000 ريالًا قطريًا في الشهر

4) 30,000 ريالًا قطريًا فأكثر

5) أرفض الإجابة

خ) هل يوجد أكثر من فرد له دخل في أسرتك المعيشية

نعم ☐ لا ☐

د) ما هي ديانتك: الإسلام / المسيحية / أخرى (يرجى تحديدها)؟

ذ) الحالة الاجتماعية (اختر الإجابة المناسبة من الخيارات الواردة أدناه):

أعزب / متزوج / مطلق / أرمل

ر) العدد الإجمالي لأفراد الأسرة المعالين داخل قطر وخارجها:

ز) المستوى التعليمي: (اختر الإجابة المناسبة من الخيارات الواردة أدناه)

أساسي (حتى الصف الخامس) / إعدادي (حتى الصف العاشر) / ثانوي (حتى الصف الثاني عشر أو ما يعادله) / جامعي / دراسات عليا / ماجستير / دكتوراه / تعليم غير رسمي / دبلوم / أستطيع قراءة وكتابة اسمي فقط / لا أستطيع القراءة والكتابة

س) منذ متى وأنت تعيش في قطر؟

1) _____ عامًا و______ شهرًا

2) طوال حياتي

تفاصيل أخرى:

أ) تاريخ اللقاء
ب) اسم المحاور
ت) اسم المشرف
ث) اسم البلدية
ج) رقم التجمع
ح) تاريخ بدء اللقاء
خ) تاريخ انتهاء اللقاء

*** نشكرك على وقتك ومجهودك الثمين ونرحب بأي اقتراحات / آراء تخص الاستبيان وتحسينه**
رقم تسلسل المشارك

معلومات إضافية للتحقق من الجودة

اسمك بالكامل:

تفاصيل الاتصال:

محل الإقامة في قطر:

اسم البلدية:

رقم التجمع:

عنوان الإقامة (سيقوم بتعبئتها جامع البيانات بنفسه)

تفاصيل أخرى:

تاريخ اللقاء:

اسم المحاور:

رقم العداد:

اسم المشرف:

تاريخ بدء اللقاء :

تاريخ انتهاء اللقاء:

معلومات اضافية

هل تود المشاركة فى اعطاء الموافقة بالتبرع بالاعضاء من احد افراد

الاسرة فى حال طلب منك المساعدة فى ذلك
